# Evaluation of an Education Programme for Introducing Bioelectrical Impedance Analysis to Neonatal Unit Staff: A Mixed Methods Study

**DOI:** 10.1111/nicc.70446

**Published:** 2026-03-13

**Authors:** D. L. McCarter, C. Morgan, L. Bray, L. Tume

**Affiliations:** ^1^ Edge Hill University, Faculty of Health, Social Care & Medicine Ormskirk UK; ^2^ Neonatal Intensive Care Unit, Liverpool Women's Hospital Liverpool UK; ^3^ Paediatric Intensive Care Unit, Alder Hey Children's NHS Foundation Trust Liverpool UK

**Keywords:** bioelectrical impedance analysis, education, fluid management, multidisciplinary team, preterm neonates

## Abstract

**Background:**

Bioelectrical Impedance Analysis (BIA) is a non‐invasive method of body composition, which may be useful in improving fluid management decisions for sick and preterm infants.

**Aim:**

To implement and evaluate an education package for the multidisciplinary team who plan to use BIA within the Neonatal Intensive Care Unit (NICU) in future research.

**Study Design:**

A mixed methods approach was used to evaluate the impact of an education package for the multidisciplinary team (MDT) working in the NICU regarding the use of bioelectrical impedance analysis (BIA). Knowledge acquisition was assessed and feedback sought regarding the content, format and delivery of the teaching.

**Results:**

There was a significant improvement in the median knowledge scores from 2.5 to 4/5 (*p* = < 0.001) after the education programme, with staff reporting a positive learning experience.

**Conclusion:**

This neonatal education package improved knowledge and was evaluated positively and could be used by other centres wishing to implement this technology.

**Relevance to Clinical Practice:**

This education package will be useful for other NICUs wanting to implement this new technology into research or clinical practice. This model of education could be altered to facilitate the introduction of any new piece of technology into any intensive care setting.

## Impact Statements

- What is known about the topic?
  ○ Bioelectrical Impedance Analysis (BIA) is a non‐invasive technology that can be used to measure body composition and total body water.
  ○ The potential application of BIA in neonatal care is evident and there is a need for contemporary research using current BIA technology within this population.
  ○ If this technology is to be incorporated into neonatal care successfully, staff must be taught how to interpret and apply the results.
  ○ Teaching within the clinical area is central to successful health professional education.
- What this paper adds?
  ○ Evidence that knowledge around BIA within the NICU was very poor at baseline.
  ○ A robustly developed education package which can increase knowledge of BIA and fluid management in the NICU.
  ○ Evidence around preferred teaching formats in a NICU.

## 1 Introduction and Background

Optimising the management of fluid provision for sick or premature babies is an integral part of successful neonatal care [1]. Accurately assessing the quickly evolving fluid status of the infant is a clinical challenge with risks for survival and/or complications [2]. Bioelectrical Impedance Analysis (BIA) is a non‐invasive method of measuring body composition and total body water (TBW) [3]. Due to an international change in licencing agreements in 2019, this technology has recently been made available for use within the neonatal population for babies as young as 23 weeks gestation [4]. A recent survey has found that despite its potential, BIA is not routinely used in clinical practice within Neonatal Intensive Care Units (NICUs) across Europe or even worldwide [5]. This education package regarding BIA described within this article is a precursor to a randomised control trial, which aims to evaluate the clinical utility of BIA when used within the NICU.

BIA is a non‐invasive, accessible and instantaneous method of measuring body composition [6]. It calculates output measures by quantifying the impedance to alternating electrical currents as they pass through an infant's body between two sets of adhesive electrodes, usually on hands and feet [7]. The electric current passes at varying rates depending upon the conductivity of the components, with fluid‐filled areas conducting electricity quickly, compared to more insulative structures such as bone and body organs [3]. Body composition measurements are calculated and produced using previously validated algorithms [8]. A recent scoping review outlined that BIA has been used within neonatal care in three categories: (1) for fluid status evaluation, (2) as a measure of adequate nutrition and growth, and (3) to validate the technology as an outcome measure in neonates [9]. Up until this time, BIA has only been used within neonatal research and not in relation to direct clinical care. With the rising survival rates for extreme preterm infants and increasing complexity of accompanying fluid management decisions [10], an exploration of this technology within the NICU clinical setting seems judicious. It is a future intention to evaluate the clinical utility of BIA when implemented into the NICU setting in a randomised control trial. Therefore, there was a need to develop an education programme around BIA for the whole multidisciplinary NICU team. No previous education packages exist (or have been evaluated) around the use of BIA within the NICU clinical. Thus, this paper describes our study to implement and evaluate a new education package.

## 2 Aim

This study aimed to implement and evaluate a BIA education programme, which was delivered over a period of 4 months (March 2024–June 2024). This education package regarding the use and application of BIA to the NICU was developed for the multidisciplinary team in a large NICU.

## 3 Design and Methods

A mixed methods approach was used to evaluate the impact of a BIA education package for the multidisciplinary team (MDT) working in the NICU. The Kirkpatric Evaluation Model (2016) underpinned this study, this model outlines four levels of evaluation of relevance to education interventions; (1) reaction, (2) learning, (3) behaviour and (4) results. This work aimed to evaluate the first two stages: reaction and learning, with the final two stages being assessed by a future RCT. The success of learning was explored by gauging the acquisition of intended knowledge and skills using self‐report pre‐ and post‐learning electronic surveys. To evaluate participants' ‘reaction’ to the learning, the project examined clinicians' views concerning the content, format, delivery and reported impact of the education programme during a World Café focus group. The project used a mixed‐methods, sequential design QUANT‐qual [11]. This study is reported in line with the CHERRIES checklist for survey reporting [12].

### 3.1 The Education Package

The package comprised of six face‐to‐face seminars lasting approximately 30 min (Table 1). The aim was for all staff to attend or view each session. Sessions were delivered face to face by the researcher (an Advanced Nurse Practitioner). Recordings were also made available via a staff NICU App. The sessions were held across 7 days of the week, at times to suit both day and night staff. Interaction was encouraged throughout the sessions, promoting an engaging learning approach; an online quiz was utilised to encourage learner engagement and to prompt learning conversations. A series of case studies and clinical scenarios were also discussed to link theory to practice [13].

**TABLE 1:** The education session names.

Session number	Session title
1	Fluid compartments in the newborn
2	Fluid provision and management in the newborn
3	Sodium and its role in the fluid management of the newborn infant
4	Bioelectrical impedance How do we use the device in the NICU?
5	Bioelectrical impedance Why should we use the device in the NICU?
6	Bioelectrical impedance Neonatal case studies

### 3.2 Setting and Sample

The study setting was a large regional NICU in Northwest England (42 cots; 12 intensive care, 12 high dependency and 18 low dependency). The NICU admits around 1000 babies a year. The sample was the NICU team consisting of 20 Neonatologists, 25 Resident Doctors, 29 Advanced Neonatal Nurse Practitioners (ANNP), 3 Allied Health Professionals (AHP), 174 Nurses and 22 Health Care Support Workers. All were eligible to attend the education sessions and participate in the study. This was a total of 273 potential participants.

### 3.3 Data Collection Tools and Methods

All participants were asked to complete one pre‐learning (Pre–survey—S1) and two post‐learning e‐surveys (Post‐surveys 1–S1 and Post‐survey 2–S2) using the Microsoft Forms platform. Individuals were assigned an ID code to facilitate pseudo anonymisation and the matching of pre‐ and post‐education survey answers. All survey instruments were developed by an expert study team to establish content validity. Prior to the start of the education package, an ANNP/member of the education team was sent the link to the pilot survey instruments. They were asked to sense check the questions and test the survey logic. The aim of the pilot test was to establish face validity. Small changes were made to the survey logic prior to the start of the study. The pre‐survey instrument (S1) was made up of seven sections, each consisting of 6–7 questions aligning with the topics covered in each teaching session. The Likert scale questions asked participants to gauge self‐assessed understanding of neonatal fluid management and BIA on a scale of very poor, poor, fair, good or excellent (1–5). The survey instrument was distributed by the NICU administration team via participants' work email and was available in the classroom prior to the start of the first session via QR code.

Two post‐learning e‐survey instruments were distributed following the final session also using same platform. The first aimed to gauge knowledge acquisition and contained a replica of the initial Likert Scare self‐assessment survey (S1). The second aimed to explore participant perceptions of the utility of the education programme, format, delivery and potential areas for improvement (S2). These e‐survey instruments were accessed either in the classroom at the end of the session via QR codes, via a QR code on a study poster in the NICU or through links sent to their work email.

#### 3.3.1 World Café

A World Café discussion was held a month following completion of the education package teaching, in the NICU seminar room. Participants were divided into four multiprofessional groups with varying levels of clinical experience and asked to answer questions. Once one set of questions had been explored, the groups moved to a different station, with different themed questions. Each group was given a flip chart to address a different given question in turn, which the next group added to. The questions were: (1) Do you feel that the information provided about Bioelectrical Impedance Analysis was sufficient? Is there anything else you feel you need to know? What could be improved? (2) Do you feel that the information provided about fluid management was sufficient? Is there anything else you feel you need to know? What could be improved? (3) How did you feel about the chosen case studies? Did these case studies help? Why or why not? Are there any other types of cases you would have liked to explore? What could be improved? (4) What are your thoughts about the format and delivery of the education package? Was the format accessible to all? Was the delivery effective? What could be improved? This method of data collection has been found to be useful to facilitate dialogue with a large group of people for exploration, verification and to facilitate learning [14]. It was led by DM and an independent neonatologist known to all the participants. The aim was to further explore the participants' perceptions of the education programme content, provision and perceived challenges for implementing BIA into practice.

World café responses were audio recorded by a Dictaphone placed on each table. The world café data presented in this paper is anonymised and reported by professional group and participant number.

#### 3.3.2 Data Analysis

Data were checked by DM and data entries were removed if the individual had not attended the teaching either on‐line or in person. For the self‐assessment survey, pre‐responses were removed if there was not a corresponding post‐teaching response.

The e‐survey data was analysed descriptively and checked for normality using Shapiro‐Wilk test in Microsoft Excel and IBM SPSS V29. As the Likert scale data was not normally distributed and involved a relatively small sample size, non‐parametric tests were used to test the difference in median self‐assessed knowledge level before and after the educational intervention [15].

The qualitative data were transcribed and coded using inductive content analysis procedures [16]. Content analysis followed these steps: data familiarisation, data reduction and grouping, and identification of concepts [17]. Data familiarisation occurred during transcription and proof reading by the lead researcher. Sections of data which illustrated points of meaning/interest were coded. The coding was checked and discussed between the lead researcher and an experienced qualitative researcher. Some codes were refined as a result. The codes were then grouped according to the key content developed from the inductive coding and in consideration of the main topics discussed within the World Café format. Quotes have been used to convey the message from within the text [18] and labelled according to the professional group and participant number.

##### 3.3.2.1 Rigour, Validity and Trustworthiness of the Data

The electronic nature of the survey instruments allowed for anonymous responses which aimed to encourage honesty. The survey instruments were pilot tested by an independent clinician to ensure they were fit for purpose, before being distributed by an independent administrative assistant. Every session of the education package was delivered by the same clinician within the same setting, using the same resources. The opportunity to participate in education package was made available to all clinical staff. The use of a statistical test was thought to enhance validity [11]. The use of the CHERRIES checklist for survey reporting supports robust reporting and trustworthiness [12].

The lead researcher acknowledges that their role as a clinician who was known to many participants may have influenced responses during qualitative data collection; however, an independent researcher was available to answer questions and provide clarification. The analysis was conducted by two members of the team to add dependability of the data. Triangulation of the world café data with data collected with survey instrument 2 aimed to add credibility to the findings [19]. The diversity of participants allowed for a range of professions and participants with differing clinical experience.

### 3.4 Ethical and Institutional Approvals

Ethical approval was obtained from the UK Health Research Authority (HRA) (337884) on 12/02/24. Participant information leaflets were circulated to all NICU staff members. Participation in the evaluation work was voluntary, and consent to participate was indicated by a tick box at the beginning of the survey instrument (S1 and S2) and the completion of a consent form prior to participation in the World Café. Survey instrument responses were pseudonymised to aid confidentiality.

### 3.5 Results

#### 3.5.1 Participants and Response Rates

There were initially 106/273 (39%) pre‐education survey responses and 64/273 (23%) post‐survey 1 responses (S1). After data cleaning, there were 42/273 (15%) paired pre‐survey and post‐survey 1 results (S1). Respondent demographics can be seen in Table 2.

**TABLE 2:** Pre‐ and post‐survey 1 participant demographics.

Professional role	Total (*n*)	Respondents (%)
Resident Doctor—ST4 or above	1	2.4
ANNP	5	12
Nurse—Team Leader	4	10
Nurse—Education Team member	2	4.8
Senior Neonatal Nurse	13	31
Neonatal Nurse	17	40
Highest qualification		
MSc	12	29
Bachelor's Degree	24	57
Diploma	5	12
A level	1	2.4
Years experience		
Over 10 years	18	43
5–10 years	11	26
1–5 years	11	26
Under 1 year	2	4.8

We received 49/273 (18%) responses to post‐survey 2 (S2). Respondent demographics can be seen in Table 3. Fifteen participants attended the World Café. They were selected using a sampling matrix to ensure a mixture of experience, grade, education and role (Table 4).

**TABLE 3:** Participant demographics post‐survey 2.

Professional role	Total *n*	Respondents %
Resident doctor—ST4 or above	1	2
ANNP	9	18
Nurse—Team Leader	1	2
Nurse—Education Team member	2	4
Senior Neonatal Nurse	18	37
Neonatal Nurse	17	35
Healthcare Support Worker	1	2
Highest qualification		
MSc	17	35
Bachelor's degree	27	55
Diploma	5	10
Years experience		
Over 10 years	20	41
5–10 years	11	22
1–5 years	15	31
Under 1 year	3	6

**TABLE 4:** World café participant's professional role.

Professional role	Total *n*	Participants %
Resident Doctor—ST4 or above	2	13
ANNP	2	13
Nurse—Team Leader	3	20
Senior Neonatal Nurse	6	40
Neonatal Nurse	2	13
Healthcare Support Worker	1	6

#### 3.5.2 Pre and Post‐Education Self‐Assessed Knowledge Acquisition

**FIGURE 1:**
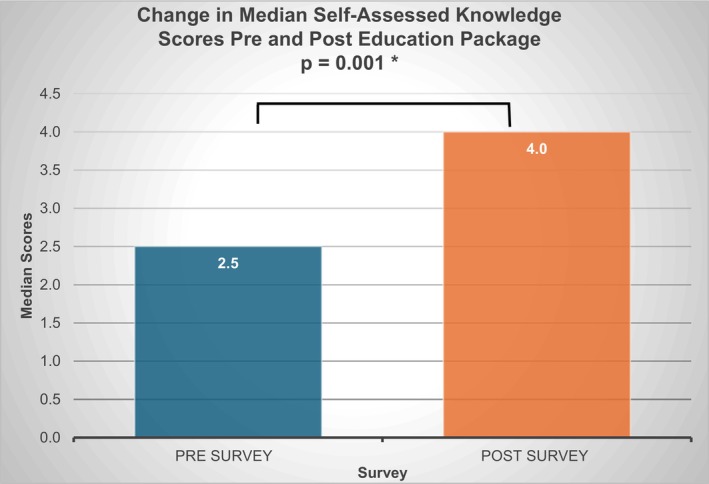
Mean participant pre‐survey and post‐survey 1 overall score.

The paired responses showed a significant increase in the median self‐assessed knowledge scores after the education package from 2.5 to 4.0/5 (*p* = 0.001) (Wilcoxon‐rank test) (Figure 1).

**FIGURE 2:**
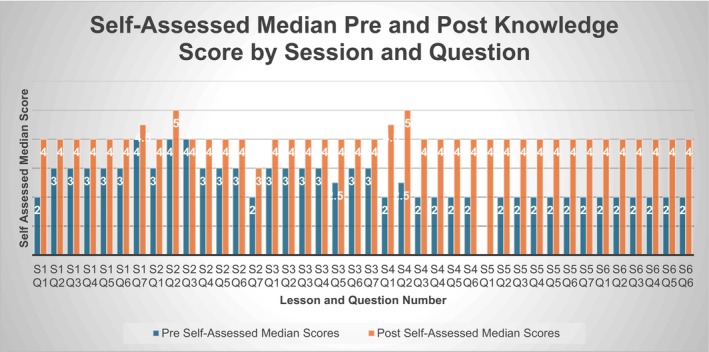
Mean participant pre‐survey and post‐survey 1 score—lessons 1–6.

This increase in median pre and post self‐assessed knowledge scores following each individual session 1–6 can be seen in Figure 2. There was an increase in self‐assessed median knowledge scores in all areas after the education, with a larger increase following the sessions which introduced completely new information and concepts; sessions 4–6 which had a focus on BIA use.

#### 3.5.3 Post‐Education Survey 2

There were 49/273 (18%) responses to the post‐survey which aimed to explore participant perceptions of the utility of the education programme (S2). In this, respondents were asked to rate the content, format and delivery of the sessions on a Likert scale of 1–5 (very poor—Excellent). An overall score for all six sessions can be seen in Table 5.

**TABLE 5:** Participant rating of the content, format and delivery of all sessions.

	Content %	Format %	Delivery %
Excellent	69	66	67
Good	21	23	22
Fair	0.34	0.34	0.34
Poor	0.34	0.34	0.34
Very poor	0	0	0

The participants have rated the content, format and delivery of the education package as mostly excellent or good. The fair and poor ratings in all areas were provided following session 1—fluid compartments. This was a physiology‐based session and included complex concepts.

#### 3.5.4 Qualitative Data Analysis

Inductive content analysis resulted in three themes reflecting the world café data: the content of the education package, the delivery of the education package, and multi‐disciplinary learning and implementing BIA learning into practice. Individual quotes are used to illustrate the concepts [18]. These are labelled by professional role and identified by number in the order that they contributed to the discussion (M; Medic, ANNP; Advanced Neonatal Nurse Practitioner, TL; Team Leader, N; Nurse and HCS; Health Care Support Worker).

#### 3.5.5 The Content of the Education Package

All participants felt that their knowledge and understanding of fluid management in the NICU and the use of BIA had improved because of participating in the sessions. Most participants discussed how the content of the education sessions had been ‘pitched at the right level’ *(ANNP 2, N5)* and it helped them ‘*learn loads’ (HCS1)* *and* *‘*reinforce what we know’ *(N4)*. One participant felt that they would have liked the information to be supplemented with ‘links if you wanted to look yourself into it – further study’ *(N3)*. And one participant reported the content as too difficult for them as ‘Quite a lot of it went over my head’ *(N2)*.

Some nurses specifically commented that they valued learning about BIA as they perceived themselves as the core and constant caregivers of babies on the NICU.

We have a core of ANNPs and Consultant – the Doctors change. They rely on us to remind them. (N4).

One of the nurses commented how the session was ‘like we used to do in the old days’ (N5) when nursing staff would have regular education sessions at hand over time. Feedback was noted that participants would have liked to have some practical experience of using the machine, perhaps using the machine on a mannikin.

The clinical applicability of the content helped staff engage in the sessions, ‘I could see the relevance of it. Not just being done for the sake of it’ *(N5)*. *‘*You can see impact on care’ *(ANNP2)*. This clinical relevance was supported using scenarios which helped ‘see it in practice and visualise it properly’ *(N6)*.

Some participants commented that they would have liked more case studies to show the application of the BIA device to a broader range of babies/clinical scenarios including ‘*extreme preterm’ (M1)*, *‘*HIE [hypoxic ischaemic encephalopathy]’ *(TL2), ‘Pre/post op’ (ANNP1), ‘gastroschisis’ (M1)* and ‘*thermoregulation’ (M1)* and a ‘round up session’ *(N3)* following the six sessions.

#### 3.5.6 The Delivery of the Education Package and Multidisciplinary Learning

Many of the participants discussed how the ‘*snappy’ (ANNP1)* and ‘*concise’ (ANNP2)*, length (30 min) of the education sessions had been a positive, as was the timing of the delivery to cover all clinical shifts, making the training ‘accessible at all times of day and night’ *(HCS1);* *‘*She (the educator) did twilight shifts, days and nights’ *(ANNP1)*.

Participants also reported the value of *sessions being* *‘*close together, so you didn't forget what had gone before’ *(TL2)* and being recorded to ‘go back and watch it’ *(N1, N2)*.

Several of the participants commented that the content was delivered with enthusiasm, confidence and with the facilitator ‘knowing what she is talking about’. *(TL3)*.

Several staff also discussed that education attendees had ‘time to answer’ *(ANNP1)* and any points were ‘re‐explained if needed’ *(TL3)*. Participants explained how they ‘loved how interactive it was’ *(ANNP1, ANNP2, TL2)* and that ‘It felt like a game’ *(N7)*, and this helped to maintain engagement. The knowledge quiz at the end required staff to use their phones to answer some questions; there were some problems encountered with ‘Internal internet problems make it frustrating’ *(N3), staff not having their phones to hand or not wanting to use a personal device (N4)*.

Participants liked the fact that. ‘Everyone was included’. *(TL1) enjoying the* chance to learn as a multidisciplinary team with a ‘mix of nurses, doctors and ANNPs’ *(N5), bringing* *‘*different ideas and opinions’. *(HCS1)*.

#### 3.5.7 Implementing BIA Learning Into Practice

Participants reported a strong desire for visual aids and resources to help in the workplace, such as ‘a laminated folder with resources in’ *(N3)* or ‘bite sized cards with criteria for use’ *(N5)*.

There were some contrasting opinions about whose role it was to use the BIA machine in practice, with some reporting ‘it was good that nurses got it as well, it is good if you are looking after the little ones that you know’. *(TL2)* and some expressing concerns about ‘my role as a nurse’ *(N3)* and whether ‘Will it take me away from my role as a carer?’ *(N3)* and a sense it was ‘more for the medical team’ *(N3)*. There were also concerns about the ‘provision of on‐going training’ *(ANNP1)* and ‘if audit was required to monitor accuracy’ *(ANNP1)*.

## 4 Discussion

To the best of our knowledge, this is the first study to report the implementation and rigorous evaluation of an education package specific to implementation of BIA in the neonatal setting. This technology has only been recently available for use in the neonatal setting, and no focused education packages exist to support its implementation. Despite this education package having a focus on BIA, the model of education discussed could be adapted for any new piece of equipment in any intensive care setting. The importance of effective implementation to enhance success is highlighted [20]. With reference to our underpinning framework, reaction and learning can be described as the two initial aspects of teaching evaluation [21].

### 4.1 Reaction to the Education Package

In terms of reaction, the education package was well received overall. In our study, this in‐person learning was appreciated by the staff and although the sessions were also available online, face‐to‐face interactions were highlighted as central to meaningful teaching interactions [22]. The opportunity for learners to collaborate and network to improve the learning experience is recommended [23].

The educator running the sessions was an ANNP and therefore might be seen as a peer mentor for many of the participants engaged with the education programme. Feedback highlighted that she was approachable, not patronising and explaining topics at an appropriate level. Shared identity and professional standing have been found to enable effective communication, enhance knowledge transfer and provide positive role modelling [24]. The status of the teacher, however, may have also had a negative effect upon uptake of the course, with only 9.4% of initial respondents being from the medical profession and only 2.4% of the sample who completed both the pre‐ and post‐learning survey (S1). Lijedahl et al. [25] suggests that social rules, norms and aspects of power can impact teaching and learning and that these can be at conflict within the medical and nursing professions.

The vast availability of the teaching sessions was highlighted as a positive when reviewed by NICU staff, with the variety of day and nighttime teaching being beneficial. The length of the teaching session was found to be acceptable; this would align with evidence that suggests meaningful clinical teaching should be brief and contain small amounts of information [22]. Members of staff voiced concerns, however, that it is difficult to concentrate on learning as they were distracted by looming clinical tasks. Workload has been found to have a negative impact upon learning [13].

Staff appreciated the relevance of the teaching to their clinical role, and this may have had a positive impact upon staff engagement. Learning is enhanced when professionals appreciate the application of the topic to their own environment [22]. The use of a quiz and other props was popular within the group, and methods such as these have been found to enhance learning [26]. Respondents reported that teacher enthusiasm had a positive impact upon their learning; this is supported by work which suggests that rapport with the educator impacts the success of learning [22].

Overall staff were complementary about the content, format and the delivery of the education package, (Table 5). For the future, participants have requested additional resources such as bite sized revision cards, specialist BIA users and reference guides. Qualitative feedback suggested that they would have liked to have used the machine practically within the teaching, and this will be incorporated into future BIA teaching.

### 4.2 Learning Within the Clinical Setting

Learner self‐assessment was used as a gauge of knowledge acquisition in the pre‐ and post‐survey (S1). This method of evaluation has been found to be appropriate in Advanced Practice Nursing programmes [27]. It must be highlighted that this education programme was for the multidisciplinary team and that the self‐assessment survey instrument was not validated. It was however tested prior to first use by an independent professional who identified possible logic problems and requested the clarification of certain terms to do with fluid spaces (TBW; total body water, ICW; intracellular water, ECW; extracellular water, IVF; intravascular fluid and EVF; extravascular fluid). The ability for learners to self‐assess accurately is called into question and the inability to link knowledge increase to actual competence must be considered [28]. Despite this, an increase in pre‐ and post‐learning self‐assessment scores cannot be denied. The first three sessions, which focused on fluid physiology and clinical management, demonstrated a smaller increase in knowledge when compared to the last three sessions, which concentrated on BIA and its application to the NICU. As the final three sessions contain information about concepts that are new for the staff, this could be said to be appropriate.

Engagement with the study programme decreased over the course of the 6 sessions with attendance at the first session being higher than at session 6 (session 1 = 120/43% session 6 = 102/37%) and engagement with survey work declining (106/39% pre‐teaching responses (pre‐survey) and 64/23% post‐teaching response (post‐survey 1)). It must be highlighted; however, that the final paired pre/post‐survey consisted of only 15% of eligible participants and a small sample size. Prolonged engagement can be troublesome within the clinical environment, with effectiveness of CPD depending upon organisational culture [23]. The importance of creating a culture that supports learners to apply their new knowledge and skills to the workplace has been highlighted [23]. One of the students had suggested that a timetable might have been beneficial to help them to understand what had gone before and what was still to be explored. Identifying and understanding proposed outcomes for learning are beneficial for teacher and student and are essential when evaluating learning [29]. It could be agreed that a transparent lesson plan might have been beneficial and improved engagement.

## 5 Limitations

There are some limitations of the study that warrant mentioning. Although developed with both content and face validity, the survey instruments used were not formally validated. Our low engagement and poor response rates may have impacted negatively upon the results. The ability to attend sessions during the working day was affected by the business and acuity of the NICU at the time of the project. Although the education materials were designed for the MDT, the lack of medical engagement may reduce the transferability of these results to the wider MDT. The researcher leading the sessions was an ANNP on the unit and therefore might be seen as a peer mentor for many of the participants who engaged with the education programme. This may have contributed to an increase in positive feedback. The ANNP was both providing the teaching resources and collecting evaluation data. Despite survey feedback being pseudonymised, this may have biased evaluations; despite these limitations, this was a robust evaluation of an education package to introduce a new piece of technology into clinical decision‐making in the NICU.

## 6 Implications for Practice

Multidisciplinary learning provided within the workplace that is interactive, relevant to practice and inclusive has been welcomed by the NICU team. This education package has been successful in educating the workforce about the use of BIA within the NICU and will facilitate the delivery of a clinical trial of the technology within this setting.

## 7 Conclusions

This is the first study to the best of our knowledge to develop and evaluate a bespoke education package around BIA for use in the clinical NICU setting. We found this multiprofessional, face‐to‐face, hybrid, interactive and accessible education package was effective in increasing self‐assessed knowledge regarding the use and application of BIA within neonatal care. Overall, the intervention was evaluated positively by the multiprofessional team. This package and educational model can be used by other NICUs wanting to implement this new device or any new piece of technology into this setting.

## Author Contributions

All listed authors have contributed to the manuscript substantially and have agreed to the final submitted version.

## Funding

There has not been any outside funding provided to support this research project.

## Ethics Statement

Ethical approval has been provided for this project—UK Health Research Authority (HRA) (IRAS ID 337884). 12/02/2024.

## Consent

This is a staff project and consent for participation and the publication of results was provided by each participant.

## Conflicts of Interest

The authors declare no conflicts of interest.

## Supporting information


**Supporting Information: S1** Pre‐survey and post‐survey 1.

**Supporting Information: S2** Post‐survey 2.

**Figure S1:** Change in median self‐assessed knowledge scores pre‐ and post‐education package. *Wilcoxon signed‐rank test.
**Figure S2:** Self‐assessed median pre‐ and post‐knowledge score by session and question.

## Data Availability

The data that support the findings of this study are available from the corresponding author upon reasonable request.
