# Supplementary material for: Evaluation of an Education Programme for Introducing Bioelectrical Impedance Analysis to Neonatal Unit Staff: A Mixed Methods Study
Source: Nurs Crit Care. 2026 Mar 13;31(2):e70446. doi: 10.1111/nicc.70446 (PMC12984483; doi:10.1111/nicc.70446)
Supplement: Supplementary file 1 — Supporting Information: S1 Pre‐survey and post‐survey 1. [file NICC-31-0-s002.docx]

**S1**

**The Bioelectrical Impedance Analysis (BIA) Education Programme Staff Evaluation Project**

Pre/Post-Education Programme Survey

Dear Colleague,

Thank you for taking part in this pre-education programme survey. By completing this survey, you are agreeing to take part in this research project.

You may download a copy of these responses for your records if you wish.

I confirm I have read the participant information sheet and understand what is being asked of me.

This survey consists of 7 sections, with 7 short Likert scale questions. It will take less than 10 mins to complete.

If you have any questions, please contact: Research@

Many Thanks

The Research Team

**Study ID**

**Please enter the ID code that has been assigned to you by the Research team:**

**Section 1: Background**

**What is your professional background?**

Medical Consultant

Medical ST4 or above

Medical ST 1 or above

ANNP

Team Leader

Education Team member

Senior Neonatal Nurse

Neonatal Nurse

Neonatal Assistant

Student

**What is you highest level of educational attainment?**

PhD

MSc

Bachelor’s Degree

Diploma

A Levels

Vocational Qualification

GCSE

None of the above

**How much experience do you have working in the NICU?**

Under 1 Year

1-5 Years

5-10 Years

Over 10 Years

**Section 2: Fluid Compartments in the New-born Infant**

**How would you rate your knowledge of:** (Likert 1-5)

Fluid compartments in an adult?

Fluid compartments in a new-born?

Homeostatic mechanisms that control fluid distribution in the new-born?

New-born postnatal adaptation and resulting fluid changes?

Clinical assessment of fluid status?

Measurements that contribute to fluid assessment?

How important do you think accurate fluid management is to our care within the NICU?

**Section 3: Fluid Management in the New-born Infant**

**How would you rate your knowledge of:** (Likert 1-5)

How preterm physiology might affect fluid management?

Routes that preterm infants can lose water from?

Clinical assessment of fluid status and how this affects fluid management?

Measurements that contribute to fluid assessment and how these affects fluid management?

Research evidence and resulting recommendations regarding fluid management?

The role of blood pressure in fluid management decisions?

The Crystalloid V Colloid discussion in the neonate

**Section 4: Sodium and It’s Role in the fluid management of the new-born infant**

**How would you rate your knowledge of:** (Likert 1-5)

Homeostatic mechanisms that control sodium distribution in the new-born?

New-born postnatal adaptation and resulting sodium changes?

The Kidney and the nephron and how these regulate sodium in the new-born?

The difference between the adult, new-born and preterm kidney?

Research evidence and resulting recommendations regarding sodium provision in the new-born?

Expected changes in sodium level in the first week of life?

Expected changes in body weight in the first week of life?

**Section 5: How do we use BIA in the NICU?**

**How would you rate your knowledge of:** (Likert 1-5)

What the Bioscan measures?

Why we would use the Bioscan in the NICU?

How to sign into the Bioscan?

The information you need to enter onto the personal info page?

How to attach the electrodes?

How to call back previous readings?

**Section 6: Why should we use BIA in the NICU?**

**How would you rate your knowledge of:** (Likert 1-5)

Expected body composition in the new-born?

How the Bioscan works?

What TBW is?

What FFM is?

What ICW is?

What ECW is?

Research evidence regarding the use of BIA in the NICU?

**Section 7: Bioelectrical Impedance Analysis used within NICU case studies?**

**How would you rate your knowledge of:** (Likert 1-5)

What information might be available from the Bioscan?

How this is viewed on the machine?

How BIA results might appear in the preterm infant?

How BIA results might appear in the term infant?

How BIA results appear in the surgical might infant?

Expected fluid trends in the new-born period?

Thank you for completing the pre-education programme survey
