# Supplementary material for: Evaluation of an Education Programme for Introducing Bioelectrical Impedance Analysis to Neonatal Unit Staff: A Mixed Methods Study
Source: Nurs Crit Care. 2026 Mar 13;31(2):e70446. doi: 10.1111/nicc.70446 (PMC12984483; doi:10.1111/nicc.70446)
Supplement: Supplementary file 2 — Supporting Information: S2 Post‐survey 2. [file NICC-31-0-s003.docx]

**S2**

**The Bioelectrical Impedance Analysis (BIA) Education Programme Staff Evaluation Project**

Post-Education Programme Survey

Dear Colleague,

Thank you for taking part in this post-education programme survey. By completing this survey, you are agreeing to take part in this research project.

You may download a copy of these responses for your records if you wish.

I confirm I have read the participant information sheet and understand what is being asked of me.

I confirm I have completed the BIA Education Programme

This survey consists of 8 sections, with 7 short answer or Likert scale questions. It will take less than 10 mins to complete.

If you have any questions, please contact: Research@

Many Thanks

The Research Team

**Study ID**

**Please enter the ID code that has been assigned to you by the Research team:**

**1: Background**

**What is your professional background?**

Medical Consultant

Medical ST4 or above

Medical ST 1 or above

ANNP

Team Leader

Education Team member

Senior Neonatal Nurse

Neonatal Nurse

Neonatal Assistant

Student

**What is you highest level of educational attainment?**

PhD

MSc

Bachelor’s Degree

Diploma

A Levels

Vocational Qualification

GCSE

None of the above

**How much experience do you have working in the NICU?**

Under 1 Year

1-5 Years

5-10 Years

Over 10 Years

**Section 2: General Questions**

Have you previously used the Bioscan in the NICU?

Yes No

Have you observed the use of the Bioscan in the NICU?

Yes No

Following the training, are you confident to: (Likert 1-5)

Take a Bioscan reading of a baby in the NICU?

If not, please describe why not?

Use Bioscan readings to inform fluid management decisions?

If not, why not?

**Section 3: Fluid Compartments in the New-born Infant**

**How would you rate the following:** (Likert 1-5)

Content

Format

Delivery

Was this session delivered in person?

Was this session viewed on-line?

Would you liked to have seen something included that was not included?

Any other suggestions for improvements?

**Section 4: Fluid Management in the New-born Infant**

**How would you rate the following:** (Likert 1-5)

Content

Format

Delivery

Was this session delivered in person?

Was this session viewed on-line?

Would you liked to have seen something included that was not included?

Any other suggestions for improvements?

**Section 5: Sodium and It’s Role in the fluid management of the new-born infant**

**How would you rate the following:** (Likert 1-5)

Content

Format

Delivery

Was this session delivered in person?

Was this session viewed on-line?

Would you liked to have seen something included that was not included?

Any other suggestions for improvements?

**Section 6: How do we use BIA in the NICU?**

**How would you rate the following:** (Likert 1-5)

Content

Format

Delivery

Was this session delivered in person?

Was this session viewed on-line?

Would you liked to have seen something included that was not included?

Any other suggestions for improvements?

**Section 7: Why should we use BIA in the NICU?**

**How would you rate the following:** (Likert 1-5)

Content

Format

Delivery

Was this session delivered in person?

Was this session viewed on-line?

Would you liked to have seen something included that was not included?

Any other suggestions for improvements?

**Section 8: Bioelectrical Impedance Analysis used within NICU case studies?**

**How would you rate the following:** (Likert 1-5)

Content

Format

Delivery

Was this session delivered in person?

Was this session viewed on-line?

Would you liked to have seen something included that was not included?

Any other suggestions for improvements?

Thank you for completing the post-education programme survey
