# Supplementary material for: Evaluation of an Education Programme for Introducing Bioelectrical Impedance Analysis to Neonatal Unit Staff: A Mixed Methods Study
Source: Nurs Crit Care. 2026 Mar 13;31(2):e70446. doi: 10.1111/nicc.70446 (PMC12984483; doi:10.1111/nicc.70446)
Supplement: Supplementary file 3 — Figure S1: Change in median self‐assessed knowledge scores pre‐ and post‐education package. *Wilcoxon signed‐rank test. Figure S2: Self‐assessed median pre‐ and post‐knowledge score by session and question. [file NICC-31-0-s001.docx]

**Change in Median Self-Assessed Knowledge Scores**

**Pre and Post Education Package**

**Figure 1 Change in Median Self-Assessed Knowledge Scores**

**Pre and Post Education Package**

*** Wilcox Rank Test**

**Self-Assessed Median Pre and Post Knowledge Score by Session̪̪̪̪̪ and Question**

**Figure 2. Self-Assessed Median Pre and Post Knowledge Score**

**by Session and Question**
